# Cross-sectional investigation of insulin resistance in youths with autism spectrum disorder. Any role for reduced brain glucose metabolism?

**DOI:** 10.1038/s41398-021-01345-3

**Published:** 2021-04-20

**Authors:** Melania Manco, Silvia Guerrera, Lucilla Ravà, Marta Ciofi degli Atti, Silvia Di Vara, Giovanni Valeri, Stefano Vicari

**Affiliations:** 1 Research Area for Multifactorial Diseases and Complex Phenotypes, Bambino Gesù, Children’s Hospital, IRCCS, Rome, Italy; 2 Department of Neuroscience, Child Neuropsychiatric Unit, Bambino Gesù Children’s Hospital, IRCCS, Rome, Italy; 3 Department of Epidemiology, Bambino Gesù Children’s Hospital, IRCCS, Rome, Italy; 4 Department of Life Sciences and Publich Health, Catholic University, Rome, Italy

**Keywords:** Diagnostic markers, Physiology

## Abstract

The autism spectrum disorder (ASD) is an etiologically heterogeneous disorder. Dysfunctions of the intermediate metabolism have been described in some patients. We speculate these metabolic abnormalities are associated with brain insulin resistance (IR), i.e., the reduced glucose metabolism at the level of the nervous central system. The Homeostasis model assessment of insulin resistance (HOMA-IR) is very often used in population studies as estimate of peripheral IR and it has been recently recognized as proxy of brain IR. We investigated HOMA-IR in 60 ASD patients aged 4–18 years and 240 healthy controls, also aged 4–18 years, but unmatched for age, sex, body weight, or body mass index (BMI). At multivariable linear regression model, the HOMA-IR was 0.31 unit higher in ASD individuals than in controls, after having adjusted for sex, age, BMI *z*-score category, and lipids that are factors known to influence HOMA-IR. Findings of this preliminary study suggest it is worth investigating brain glucose metabolism in larger population of patients with ASD by using gold standard technique. The recognition of a reduced glucose metabolism in some areas of the brain as marker of autism might have tremendous impact on our understanding of the pathogenic mechanisms of the disease and in terms of public health.

## Introduction

Autism spectrum disorder (ASD) is a neurodevelopmental disorder characterized by early onset and persistent deficits in social communication and interaction, and in several areas of functioning owing to restricted and repetitive patterns of behavior, interests, or activities. ASD affects ~1 in 54 children in the United States, placing this condition as one of the most common neurodevelopmental disorders (https://www.cdc.gov/mmwr/volumes/69/ss/ss6904a1.htm?s_cid=ss6904a1_w).

In families, the relative recurrence risk of ASD increases with increasing genetic relatedness, varying from 10.3 for full siblings to 2.0 for cousins^1^. Hundreds of gene variants, many of them related to conditions other than autism, have been described in patients with ASD. Between 10 and 30% of the patients with ASD have rare de novo or inherited variants that are considered to be causal^2^. Nonetheless, ASD is an etiologically heterogeneous disorder and factors other than genetics (i.e., environment, impaired immune responses, mitochondrial dysfunction, and neuro-inflammation, etc.) act synergistically^3^ and converge every so often on common biological pathways that represent “core” mechanisms for the disease development^4^. These pathways cover a large segment of genetic variations and result phenotypically in the wide spectrum of ASD symptoms^4^. Many individuals with ASD present metabolic features^3^, i.e., low-grade inflammation, enhanced oxidative damage, lipid oxidation prevailing over glucose oxidation with high levels of lactate and low carnitines, high levels of branched amino acids (BCAAs), all of these are associated with peripheral insulin resistance (IR)^5^. Peripheral IR is the reduced ability of insulin to stimulate cell glucose uptake mainly in the muscle and inhibit hepatic glucose production^6^.

In patients with obesity and T2D, peripheral IR seems paralleling brain IR, i.e., the reduced glucose metabolism in the central nervous system (CNS). Brain IR has been associated with reduced executive functioning^7^, inhibitory control, and cognitive flexibility in young patients with obesity^8^ and implied in the pathogenesis of dementia in adults suffering T2D or Alzheimer disease^9^.

The brain utilizes ~25% of the total body glucose through glycolysis and mitochondrial oxidative phosphorylation to produce energy, to maintain neurotransmission and neuronal potential, and to prevent excitotoxicity^10^. Under normal conditions, the capacity to transport glucose into the brain exceeds the brain’s energy requirement by twofold to threefold. Glucose transport is achieved by the coordinated activity of glucose transporters (GLUTs and sodium-glucose transporter, SGLTs) on the capillary endothelium and plasma membrane of astrocytes, oligodendrocytes, and neurons. GLUTs represented in the brain are GLUT1, GLUT2, GLUT3, GLUT4, GLUT8, and SGLT1, with GLUT4 and GLUT8 in the hippocampus responsive to the insulin action. GLUT4, which is located on the surface of neurons, is mobilized as a direct response to sustained synaptic activity and membrane translocation of GLUT4 is likely insulin dependent. So brain IR is characterized by reduced neuronal glucose uptake^10^. Any alteration to neuronal glucose metabolism, largely supported by mitochondria, would affect neuronal function. Likewise, hampered mitochondrial function would lead to the production of oxidants that impair glucose utilization^11^. Interestingly, also high concentrations of D-fructose causes central neuronal IR and promotes memory impairment in animal models through a mechanism that depends on GLUT5^12^.

In this frame, brain IR impairs glucose utilization in the hippocampus, cortex, Purkinje cells in the cerebellum, vestibular nucleus in the medulla oblongata, in ependymal cells along the cerebral ventricles.

We hypothesize that brain IR is one of the core mechanisms clinically detectable in some ASD patients. Peripheral IR and metabolic perturbations might be or be not associated to brain IR and metabolic disturbances in the CNS.

In studies investigating the association of IR with cognitive impairment^8,9^, the homeostasis model assessment of IR (HOMA-IR), i.e., the ratio between the product of fasting glucose per insulin divided per a certain factor^13^, has been used representing a convenient estimate of both peripheral and brain IR. The HOMA-IR is a suitable proxy of peripheral IR in population studies in which measure of glucose sensitivity by gold standard techniques (i.e., the euglycemic hyperinsulinemic clamp or the intravenous glucose tolerance test) is unfeasible. The HOMA-IR, indeed, was found to have good correlation with measures of peripheral insulin sensitivity from the clamp or the intravenous test^14^. As to measurement of brain IR, the gold standard technique is the estimation of glucose metabolism by the 18-fluorodeoxyglucose positron emission tomography (^18^F-FDG PET). The radiotracer ^18^F-FDG is analogous to glucose and provides an index for the first step of the cellular glycolytic pathway^15^. PET studies provided the significant association between higher HOMA-IR and reduced global glucose metabolism in the brain, suggesting that HOMA-IR reflects to some extent simultaneously peripheral and central glucose homeostasis^16,17^.

HOMA-IR is an estimate of IR in fasting condition and a proxy of hepatic gluconeogenesis^6,18^, which in turn is regulated by insulin action in the CNS^19,20^.

IR is associated with an overproduction of insulin to keep glucose plasma levels within normal range in high-carbohydrate diets. Brain homeostasis is preserved from exposure to excessive insulin levels since its passage across the blood-brain barrier occurs thru a saturable transporter and uptake of glucose is largely independent of insulin in the CNS. Nevertheless, insulin exerts in the CNS important metabolic (i.e., regulation of hepatic neoglucogenesis and catabolism of BCAAs; appetite control; neuro-inflammation) and mitogenic growing undertakings (i.e., neuronal survival, synaptic maintenance, dendritic arbor development, neuronal circuitry formation)^19,20^.

Peripheral hyperinsulinemia, nonetheless, stimulates the synthesis of plasminogen activator inhibitor-1, the most potent inhibitor of tissue plasminogen activator (tPA) that, in turn, catalyzes important neuronal processes, such as cleavage of brain-derived neurotrophic factor (BDNF) precursor to anti-apoptotic mBDNF, proteolysis of vascular endothelial factor, N-methyl-D-aspartate receptor activation, and regulation of dopamine release. As a result, tPA is involved in synaptic remodeling, neuronal plasticity, cognitive, and emotional processing^21^.

Since many antipsychotic medications worsen IR and therefore make ASD patients prone to develop obesity, altered glucose metabolism and dyslipidemia^22^ that in turn worsen IR in a vicious cycle, we estimated HOMA-IR in a population of patients with newly diagnosed ASD and naïve to any pharmacological treatment and healthy youths.

## Patients and methods

We enrolled 60 young outpatients aged 4–18 years consecutively selected from those newly diagnosed with ASD at the Child and Adolescence Neuropsychiatric Unit of the Bambino Gesù Hospital between October 2017 and May 2018.

As mean of comparison, a sample of 240 subjects was randomly drawn from the “Bambino” study population (*N* = 2573), enrolled between July 2012 and 2013, with the aims at identifying genetic determinants of glucose homeostasis and drawing distribution of HOMA-IR in Italian youths. The Bambino cohort is representative of the general Italian population of healthy children and adolescents^23^.

Patients with ASD and participants to the “Bambino” study underwent anthropometric measurements, laboratory evaluation of fasting glucose and insulin, lipid profile, and liver function tests following same procedures and protocols. Patients underwent also testing of blood amino acids (AAs) and genetics, psychiatric, and psychological evaluations.

The study was done in accordance with the Declaration of Helsinki of 1975 and approved by the Ethical Committee of the Bambino Gesù Hospital (ref. 1043-OPBG-2015). Informed consent was obtained from parents or responsible guardians.

### Clinical and biochemical assessment

Weight and height were measured using standard procedures. The *z*-score of body mass index (BMI) was calculated using WHO reference values and is given as standard deviation score (SDS)^24^. Overweight was defined as BMI ≥85th (1.036 SDS) and <95th (1.645 SDS) percentiles, and obesity as a value of BMI ≥95th (1.645 SDS) percentile for gender and age. Patients and participants to the “Bambino” study were asked to refrain from intensive physical activity in the 3 days prior to the study and were prescribed a standardized diet. Two fasting blood samples were drawn 5 min apart after 8–12 h fast and the value of HOMA-IR was calculated on the average levels of fasting glucose and insulin^13^. In this study, IR was defined as HOMA-IR ≥ 2.5 unit. This value corresponds to the ≥90th of HOMA-IR for any class, age, and sex in the normal weight healthy population and it is generally considered a reliable cut-off point of IR in pediatric population studies^23^.

Fasting glucose, total cholesterol, high-density lipoprotein (HDL) cholesterol, triglycerides, alanine aminotransferase, and gamma-glutamyl transferase were measured by using commercial methods (ADVIA 1800 Chemistry System, Siemens Healthcare Diagnostic, Deerfield, IL). Serum insulin was analyzed by a chemiluminescent immunoassay method (ADVIA Centaur XP Immunoassay System; Siemens Healthcare Diagnostic, Deerfield, IL). Dyslipidemia was diagnosed in the presence of at least one of the following conditions: value of cholesterol and/or triglycerides higher than the 95th percentile and/or HDL cholesterol lower than the 5th percentile for age and sex^25^.

In patients with ASD, serum AAs were measured by liquid chromatography–mass spectrometry; copper was estimated by atomic absorption spectroscopy (An Analyst 300 Perkin Elmer atomic absorption spectrophotometer equipped with a graphite furnace platform HGA 800). Ceruplasmin levels were estimated by immunoturbidimetric assays (Horiba ABX); serum was mixed with the purified immunoglobulin fraction of, respectively, a rabbit anti-human transferrin antibody solution and a rabbit anti-human ceruloplasmin antiserum, containing 15 mmol/l NaN_3_ as stabilizer. The resulting immune complexes were measured by turbidimetry. Plasma homocysteine levels were determined by high-performance liquid chromatography^26^.

### Genetic testing

Array-comparative genomic hybridization was performed in all the subjects with ASD^27^ by using the Agilent Human Genome 4 × 180 K CGH microarrays (Agilent Technologies, Santa Clara, California, USA), following the Agilent Oligonucleotide Array-Based CGH protocol for Genomic DNA analysis (Version 6.2.1, February 2010). The array images were scanned using the Agilent scanner and elaborated by Feature Extraction software v.10.5 and Agilent Genomic Workbench Lite Edition v.7.4 (UCSC Human Genome Browser hg19). Chromosome aberrations were calculated by ADM2 algorithm. Aberrations were investigated if larger than 100 Kb in size and involving at least one known gene.

### Neuropsychiatric evaluation

ASD was diagnosed according to the *DSM-5* criteria by trained pediatric neuropsychiatrists (S.G., G.V., S.V.)^28^. Based on the patient’s collaboration and language development, cognitive development was assessed by the Intelligence Quotient (IQ) obtained from the Wechsler Intelligence Scale for Children Fourth Edition^29^, the Leiter-3^30^, or the Raven’s Colored Progressive Matrices^31^. The Griffiths III^32^ was administered when a child failed to complete the Leiter scales because of his/her reduced attentional resources, by providing general quotient (GQ). The severity of ASD symptomatology was assessed by administering the Autism Diagnostic Observation Schedule 2nd edition (ADOS-2)^33^ that provides a total score and a comparison score ranging from 1 to 10.

### Statistical analysis

Based on HOMA-IR distribution in the “Bambino” study population^23^, and presumed a difference of 0.5 unit between healthy (HOMA-IR = 1 ± 0.96) and ASD (HOMA-IR = 1.5 ± 1.4) youths, a sample size of 60 patients with ASD and 240 controls was required (alpha 0.05; power 0.90). Univariable linear regression was used to identify single determinants of HOMA-IR levels; in order to assess the simultaneous effect of those determinants on HOMA-IR level, while adjusting for possible confounding, a multivariable linear regression analysis was performed. In the whole population, variables included in the models were sex, ASD status, age, BMI *z*-score category, and lipids. In the ASD group genetics, serum AAs and lipids were included. Analyses were performed with Stata 14.1 (StataCorp LLC, College Station, TX). A *p* value < 0.05 was considered statistically significant.

## Results

Sex distribution, age, body weight, and BMI were significantly different (*p* ranging from 0.001 to <0.0001) between ASD and healthy youths. Table 1 shows values of anthropometrics, clinical, and biochemical variables of youths with ASD and controls. Figures of patients and controls with HOMA-IR ≥ 2.5 unit were reported. Genomic hybridization revealed causative genetic mutation in 4 (7%) ASD patients. Neuropsychological scores were as follows: Griffiths III-GQ 56.2 ± 14.1; IQ 83.6 ± 20; ADOS-2 total score 13.2 ± 5.3; ADOS-2 Comparison score 6.1 ± 1.6.

**Table 1:** Baseline characteristics of patients and controls.

	ASD subjects (*N* = 60)	Control subjects (*N* = 240)	*p*
Sex (M/F)	49/11 (82/18%)	126/114 (52.5/47.5%)	<0.001
Age (years)	10 ± 1.5	17 ± 3	<0.001
Body weight (kg)	38 ± 17	30 ± 15	<0.001
Height (cm)	137 ± 22	128 ± 24	0.01
BMI (kg/m^2^)	19 ± 5	18 ± 4	<0.001
BMI *z*-score (SDS)	0.67 ± 1.85	0.28 ± 1.43	0.08
Weight category			0.128
Normal weight	27 (45%)	137 (56%)	
Overweight	22 (37%)	59 (25%)	
Obese	11 (18%)	44 (18%)	
Fasting glucose (mg/dl)	84.5 ± 8	80 ± 8	<0.001
Fasting insulin (µUI/ml)	9 ± 6	6.5 ± 5	<0.001
HOMA-IR (dimensionless)	2.0 ± 1.5	1.3 ± 1.0	<0.001
HOMA-IR > 2.5	12 (20%)	13 (5.4%)	<0.001

*p* refers to the statistical significance at the *t*-test for unpaired continuous data or at the Chi square test or categorical data.

*ASD* autism spectrum disorder, *BMI* body mass index.

Table 2 reports mean values of serum metabolites of patients with ASD. Three patients (5%) had values of aminobutyrate above the normal range, six (10%) had high glycine levels, six (10%) had high valine levels, and seven (13%) had high lactate levels. Acetyl carnitine was below the normal range in six patients (10%).

**Table 2:** Mean values of serum metabolites in ASD patients.

Metabolite	Mean value ± standard deviation	Reference range
Aminobutyrate (µm/l)	21.1 ± 6.2	10–40
Ceruplasmin (mg/dl)	26.5 ± 5.3	20–60
Copper (µg/dl)	84.6 ± 15.8	80–180
Creatinine (µm/l)	60 ± 19	16–93
Glycine (µm/l)	270 ± 47.6	120–340
Homocysteine (µm/l)	10.9 ± 7.8	4–13
Isoleucine (µm/l)	63.5 ± 13.7	30–100
Lactate (µm/l)	1.5 ± 1.0	0.60–2.30
Leucine (µm/l)	127.6 ± 23	50–130
Methionine (µm/l)	24.2 ± 6.2	10–50
Phenylalanine (µm/l)	53.5 ± 10.3	30–80
Serine (µm/l)	145 ± 19.4	35–190
Tryptophan (µm/l)	13.2 ± 4.8	5–40
Valine (µm/l)	257 ± 51	150–320

Table 3 describes predictors of HOMA-IR in the whole population and ASD patients. HOMA-IR was significantly increased by 0.55 units for the ASD status; 0.17 units for year of age, and 0.38 units for being overweight/obese vs. normal weight. Modeling together these variables, in the final model HOMA-IR increased by 0.31 unit for the ASD status, by 0.47 unit for being overweight/obese vs. normal weight and by 0.17 unit for year of age.

**Table 3:** Predictors of HOMA-IR.

	Univariate analysis			Multivariate analysis		
	Coefficient	95% CI	*p*	Coefficient	95% CI	*p*
Whole population (*N* = 300)						
ASD vs. controls	0.55	0.21–0.88	**0.001**	0.31	0.02–0.61	**0.040**
Sex (male)	0.07	−0.01–0.35	0.618	0.15	−0.09–0.39	0.215
Age (years)	0.17	0.14–0.20	**<0.001**	0.17	0.14–0.20	**<0.001**
BMI *z-*score classification: normal weight (ref.)						
Underweight	−0.34	−0.70–0.02	0.061	−0.19	−0.50–0.12	0.230
Overweight/obese	0.38	0.06–0.69	**0.020**	0.48	0.20–0.74	**0.001**
ASD subjects (*N* = 60)						
Sex (male)	0.40	−0.61–1.42	0.42	0.22	−0.57–1.00	0.580
Age (years)	0.20	0.11–0.31	**<0.001**	0.18	0.08–0.27	**<0.001**
BMI *z-*score classification: normal weight (ref.)						
Overweight/obese	0.47	−0.39–1.33	0.274			
Valine	0.11	0.003–0.018	**0.004**	0.01	0.001–0.01	**0.031**
Isoleucine	0.04	0.17–0.07	**0.002**			
Leucine	0.02	0.002–0.04	**0.030**			
Triglycerides	0.02	0.01–0.03	**0.001**	0.02	0.01–0.03	**0.001**

*ASD* autism spectrum disorder, *BMI* body mass index, *HOMA-IR* homeostasis model assessment of insulin resistance.

Bold values highlight predictor of HOMA-IR which were statistically signifcant.

In the ASD group, age, BMI classification (normal weight/overweight/obesity), values of BCAAs (i.e., valine, isoleucine, leucine) and triglycerides levels were statistically significant predictors of HOMA-IR. In the final multivariable model age, and values of valine and triglycerides remained predictors of HOMA-IR (Table 3).

## Discussion

In this exploratory investigation, we tested the hypothesis that young individuals with ASD have higher HOMA-IR than peers without. We found that HOMA-IR is significantly increased by 0.31 unit for the ASD status, when an unconditional multivariable linear regression model was fitted in order to adjust for confounders and other determinants.

### BCAAs

Of the 60 patients with ASD, 28 (47%) had elevated BCAAs, low acetyl carnitine levels or other metabolic abnormalities. HOMA-IR was significantly correlated with levels of BCAAs.

The BCAAs, leucine, isoleucine, and valine, make up approximately a third of essential AAs. They are involved in cellular signaling and modulation of metabolic processes including glucose homeostasis and energy balance^34^. Their homeostasis seems crucial for the neuronal health with either low or high concentrations likely affecting neuronal function. Excessive amounts of BCAAs are toxic to the CNS via a number of different mechanisms, as evidenced from the neuropathology associated with the maple syrup urine disease^35^. Disruption of BCAA levels by branched chain ketoacid dehydrogenase kinase mutations was found to have extensive implications for survival and function of several neuronal circuits and be causative of autism, epilepsy, and intellectual disability in families of consanguineous cases^35^. Since gut bacteria produce a higher proportion of BCAAs relative to the other AAs, BCAAs might link gut dysbiosis and increased risk of ASD^36^. ASD children were shown to have an abundance of gut microbiota synthesizing BCAAs, like Bacteroides vulgatus and Prevotella copri, and a decreased number of species positively associated with insulin sensitivity, such as Bacteroides fragilis and Akkermansia muciniphila^36^. As to IR, its severity correlated with raised levels of BCAAs in patients with obesity and T2D^5^. Such significant association can be caused either by increased enrichment of BCAA-producing bacteria in the gut or by disrupted hypothalamic insulin signaling that causes decreased hepatic BCAA catabolism^37^. If altered gut microflora has with no doubt a pivotal role in the pathogenesis of ASD causing high levels of BCAAs^38^, we cannot exclude that a disruption of the insulin signaling in the CNS can contribute altering the BCAA metabolism of ASD patients. In any case, elevated BCAAs levels worsen IR.

### Mitochondrial dysfunction, carnitine system, and lactate

Our data suggest that brain IR is seen in some of the patients with ASD, possibly impairing mitochondrial functioning. Altered fatty acid metabolism^3,39^; impaired carnitine biosynthesis^40,41^; some mitochondrial dysfunctions that result in altered levels of lactate or carnitine metabolites^42,43^ are some of the abnormalities described in patients with ASD. In our series, lactate was high and acetyl carnitine low in some patients, which is consistent with dysfunctional activity of the mitochondrion.

Altered fatty acid metabolism is pivotal in the pathogenesis of IR. In the IR status, excessive availability of fats causes increased fatty acid oxidation and decreased glycolysis and glucose oxidation^6,40^. The carnitine is substrate of the carnitine acetyltransferase that has an essential role to rescue glucose oxidation, being able to switch energy substrate preference from fatty acid oxidation to glucose oxidation. In the brain there is a continuous need of energy, most of which is produced from glucose by oxidative phosphorylation in mitochondria, complemented by aerobic glycolysis in the cytoplasm. When glucose levels are limited, ketone bodies generated in the liver and lactate become important energy substrates for the brain^10^.

It is worth reminding that neonatal hypoglycemia going undetected or refractory neonatal hypoglycemia may cause transitory mitochondrial dysfunction that can harm the neonatal brain^44^.

### Brain glucose metabolism and altered neuronal connectivity

In patients with ASD, HOMA-IR might reflect reduced glucose metabolism in some brain regions as observed in healthy individuals and patients affected by other neurological diseases^15,16^.

Glucose uptake in neurons is driven by their energy demand, not by the glucose plasma levels, which means that hyperglycemia does not increase glucose uptake in neurons^10^. Reduced glucose uptake might parallel disturbed connectivity observed in patients with ASD. Individuals with ASD have mostly lower connectivity (or hypo-connectivity) between distant brain regions (such as the frontal and parietal lobes) and increased connectivity (or hyper-connectivity) between local brain regions (such as within the frontal lobe) compared with typically developing individuals^45^. Study of brain glucose metabolism by ^18^F-FDG-PET and functional magnetic resonance imaging scans found in these subjects decreased metabolic rates of glucose metabolism in the parietal lobe, frontal premotor, and eye-fields areas, and amygdala; and increased in the posterior cingulate, occipital cortex, hippocampus, and basal ganglia, with metabolic abnormalities also in the social brain^46^.

### Obesity and IR

In our series, 14 out of 60 patients (24%) were overweight and eight (14%) were obese. In this perspective, programming of altered brain glucose metabolism and insulin sensitivity might be factors favoring onset of obesity and metabolic abnormalities in these individuals. So far, youths with ASD have been deemed vulnerable to develop these abnormalities mostly by virtue of the complex behavioral, physical, and psychosocial problems that they experience and the use of obesogenic drugs (i.e., mood stabilizers, antipsychotics, antiepileptic drugs, and selective serotonin reuptake inhibitors)^17^. It cannot be ruled out altered programming of the circuitries regulating reward, feeding, and energy expenditure in individuals with ASD as seen in offspring to mothers with obesity and T2D that would increase their risk of obesity.

## Limitations and strengths

We are aware of the several caveats of this investigation that was definitely exploratory: cross-sectional design; small sample size; estimation of HOMA-IR instead of gold standard estimates of peripheral and central IR; lack of data on cognitive function and serum AAs in individuals with no ASD; no control of long-term dietary habits that might have influenced HOMA-IR values in ASD patients; no measurement of postprandial insulin values.

On the other hand, the enrollment of patients with neodiagnosis of ASD and naïve to any treatment was a strength of the investigation. Many psychiatric medications influence intermediate metabolism leading to high triglycerides, weight gain, and other dysmetabolic features that, in turn, are associated with high HOMA-IR.

### Metabolic intervention to improve mental symptoms of ASD and future directions

Reduced glucose metabolism could be the marker of an energy gap affecting the mitochondrion that develops during the gestation and persists after birth. Once glycolysis is impaired, the brain energy deficit cannot be corrected by simply increasing blood glucose concentration. Diet intervention, exercise, and drugs able to ameliorate brain energy metabolism and/or to reduce insulin levels might be useful to improve mental health in patients with ASD. Dietary-derived ketones might be convenient alternative brain energy source. The establishment of a metabolic ketosis favors stabilization of insulin levels and amelioration of the brain energy gap by bypassing glycolysis and providing acetyl-CoA to enter the tricarboxylic acid cycle directly^10^. Similarly, physical exercise normalizes fasting and post-meal insulin levels. Administration of metformin, an insulin sensitizer agent, early in life has been found to ameliorate social approach deficits, repetitive grooming and to reduce marble burying in a mouse model of ASD^47^.

Intranasally administered insulin enters the brain directly via olfactory neurons enabling the amelioration of CNS glucose metabolism while minimizing systemic hypoglycemia^48^. More importantly, insulin might work efficiently thanks to its mitogenic properties. In six children with 22q13 deletion syndrome, 1-year intranasal insulin treatment led to the significant amelioration of gross and fine motor activities, nonverbal communication, cognitive functions, and autonomy^48^. The syndrome is characterized by impaired dendritic spine formation and synaptic signaling owing to the haploinsufficiency of the scaffold protein SH3 and multiple ankyrin repeat domains (SHANK3) located at axon terminals and postsynaptic densities of neurons. In these syndromic children, beneficial effects of insulin were likely due to its capacity to upregulate another postsynaptic scaffolding protein, postsynaptic density-95, in rat neurons that compensates for the deficient SHANK3 activity^48,49^.

## Conclusions

We tested and confirmed the hypothesis that youths with ASD have in average higher HOMA-IR than those without ASD regardless of their BMI and pharmacological treatment. Studies are needed to understand whether increased HOMA-IR reflects a hampered central glucose metabolism and this feature correlates with neuropsychiatric symptoms.

Our study must be intended as provocative attempt at investigating autism as “metabolic” condition in which IR is in some patients the marker of more profound metabolic disturbances that deserve investigation toward a more personalized medicine approach. The observations discussed herein should help us move in the direction of this important goal.
